# IgA Antibodies to Bovine Serum Albumin in Adult Patients with Celiac Disease

**DOI:** 10.3390/ijms26114988

**Published:** 2025-05-22

**Authors:** Elena Savvateeva, Marina Yukina, Nurana Nuralieva, Svetlana Bykova, Ivan Abramov, Vera Polyakova, Natalia Bodunova, Maxim Donnikov, Lyudmila Kovalenko, Elena Mazurenko, Elizaveta Pavlova, Elena Kulagina, Ekaterina Troshina, Dmitry Gryadunov

**Affiliations:** 1 Engelhardt Institute of Molecular Biology (EIMB), Russian Academy of Sciences, 119991 Moscow, Russia; pavlova.elizaveta@phystech.edu (E.P.); elenka176@yandex.ru (E.K.); grad@biochip.ru (D.G.); 2 Endocrinology Research Centre, Ministry of Health of Russia, 117292 Moscow, Russia; kuronova@yandex.ru (M.Y.); nnurana@yandex.ru (N.N.); troshina@inbox.ru (E.T.); 3 The Loginov Moscow Clinical Scientific Center, 111123 Moscow, Russia; s.bykova@mknc.ru (S.B.); abriv@bk.ru (I.A.); v.polyakova@mknc.ru (V.P.); n.bodunova@mknc.ru (N.B.); 4 Department of Children’s Diseases, Medical Institute of Surgut State University, 628400 Surgut, Russia; donnikov@gmail.com (M.D.); kovalenko_lv@surgu.ru (L.K.); 5 Research Institute of Internal and Preventive Medicine—Branch of the Institute of Cytology and Genetics, Siberian Branch of Russian Academy of Sciences, 630089 Novosibirsk, Russia; poltorackayaes@gmail.com

**Keywords:** IgA, serum albumin, celiac disease, multiplex immunoassay, food immunoreactivity, interference in immunoassay

## Abstract

This study investigated the IgA antibodies targeting bovine serum albumin (BSA) in 27 adult celiac disease (CD) patients adhering to a gluten-free diet (GFD), compared to 123 controls (including individuals with autoimmune disorders, those with gastrointestinal cancers, and healthy donors). Serum samples were evaluated using a multiplex assay based on a microarray comprising 66 immobilized antigens, including autoantigens associated with autoimmune diseases, different albumins, cytokines, and inflammatory markers. Elevated IgA-BSA levels were detected in 22% of CD patients versus 3.25% of controls. IgA-BSA did not cross-react with milk proteins like casein, β-lactoglobulin, and γ-globulin, nor with autoantigens and human albumin, ruling out autoimmunity against self-proteins. The observed cross-reactivity with porcine albumin suggests that antibodies target epitopes shared by bovine and porcine albumin. Increased IgA-BSA levels may interfere with immunoassays performed using BSA as a stabilizer, necessitating protein-free buffers to avoid false results when testing CD patients. Elevated IgA-BSA levels may reflect ongoing gut barrier dysfunction in CD patients on a GFD, allowing dietary proteins like BSA to trigger immune responses. This study identifies a novel immune response in CD patients on a GFD, emphasizing the need for tailored diagnostic approaches (BSA-free assays) and further research into the clinical and dietary implications of IgA-BSA elevation.

## 1. Introduction

Celiac disease (CD) is an autoimmune disorder characterized by damage to the small intestine. Gliadin, the alcohol-soluble fraction of gluten proteins in cereals, contains the factor that is toxic to CD patients. Gliadin is a substrate for the calcium-dependent enzyme tissue transglutaminase 2 (TGM2), and the resulting complex of gliadin with TGM2 is an antigen that induces the production of autoantibodies [1]. The global prevalence of CD is estimated at 0.5–1%, varying by geographic region and population dietary habits [2]. Recent population studies have made it possible to estimate the number of undiagnosed cases: of 12,000 people screened, 1.47% were found to have CD, while 75% of patients were previously undiagnosed [3].

A lifelong gluten-free diet (GFD) remains the cornerstone of CD treatment [2]. However, some patients exhibit no symptomatic improvement despite strict GFD adherence [4]. And in some patients following a GFD, symptom relief may occur, but small intestinal mucosal damage (e.g., villous atrophy) persists [5,6]. Villous atrophy and mucosal damage in the small intestine can impair protein absorption. This may result in incomplete protein digestion, allowing intact or partially digested proteins (antigenic fragments) to enter the bloodstream. Such antigenic exposure can trigger an immune response, including antibody production. Notably, circulating antibodies against food proteins are detectable in both healthy individuals and in patients with gastrointestinal pathologies, as demonstrated in prior studies [7,8]. In particular, emerging evidence confirms IgG reactivity to dietary antigens in non-allergic healthy individuals [9]. Among other classes of antibodies, the immune mechanisms of the induction and function of food-specific serum IgA remain poorly understood [10].

Molecular mimicry between food antigens (notably bovine serum albumin, BSA) and host proteins has been proposed as a potential driver of autoimmune pathogenesis [11]. Early studies explored anti-BSA antibodies in type 1 diabetes mellitus (T1D), theorizing cross-reactivity between milk proteins and pancreatic antigens [12,13]. While initial claims of direct mimicry remain unconfirmed [14], recent evidence suggests that elevated cow milk antibody titers may signal heightened susceptibility to islet autoimmunity [15]. Some research has been conducted on IgG antibodies against BSA in cancer patients, but the results were not different from those of the healthy group and were not associated with any detectable clinical events [16]. Among non-autoimmune diseases, elevated levels of IgG BSA antibodies were found in children with Down syndrome [17]. In addition, a mechanism involving modified cationic bovine serum albumin and antibodies targeting it has been reported in the development of membranous nephropathy in children [18].

The role of antibodies targeting BSA in celiac disease has been explored in several studies. Kenrick and Walker-Smith detected anti-BSA antibodies via immunodiffusion in 6 of 16 untreated CD children, though immunoglobulin classes were not identified [19]. Follow-up analyses after GFD initiation did not reassess BSA reactivity. Falchuk and Isselbacher reported anti-BSA antibodies in five of five untreated CD patients, with elevated titers predominantly being IgG (and occasionally IgA) [20]. Similar reactivity was noted in ulcerative colitis (28 of 30 patients), Crohn’s disease (30 of 35 patients), and healthy controls (12 of 28). Kemp et al. observed elevated IgA1 anti-BSA levels in seven of eleven CD children on a GFD and four of six children on gluten-containing diets, versus three of fifty-six healthy adults and children [21]. Hvatum et al. found minimal IgG/IgA anti-BSA activity in adults with untreated CD (*n* = 14) and disease controls (*n* = 10) [22]. Recently, Rodríguez-Juan et al. identified elevated IgG anti-BSA in non-diabetic CD children on a GFD (*n* = 30) and in T1D patients with celiac-related antibodies (*n* = 17) compared to T1D patients without celiac antibodies (*n* = 63), patients with autoimmune thyroiditis (*n* = 13), and healthy donors (*n* = 45) [23]. This led to the hypothesis that IgG anti-BSA may reflect compromised epithelial barrier integrity.

While prior studies have explored serum immunoreactivity to BSA in celiac disease, these investigations primarily focused on pediatric and untreated cases. To address this research gap, the present study aimed to characterize the IgA antibody response to BSA in adult CD patients adhering to a GFD and to compare IgA and IgG reactivity across high-homology mammalian albumins, such as BSA, porcine albumin (PoSA), and human albumin (HSA). Particular emphasis was placed on evaluating IgA to BSA cross-reactivity and assessing interference in antibody assays caused by high levels of anti-BSA antibodies.

## 2. Results

### 2.1. IgA Antibody Response to BSA

**Figure 1:**
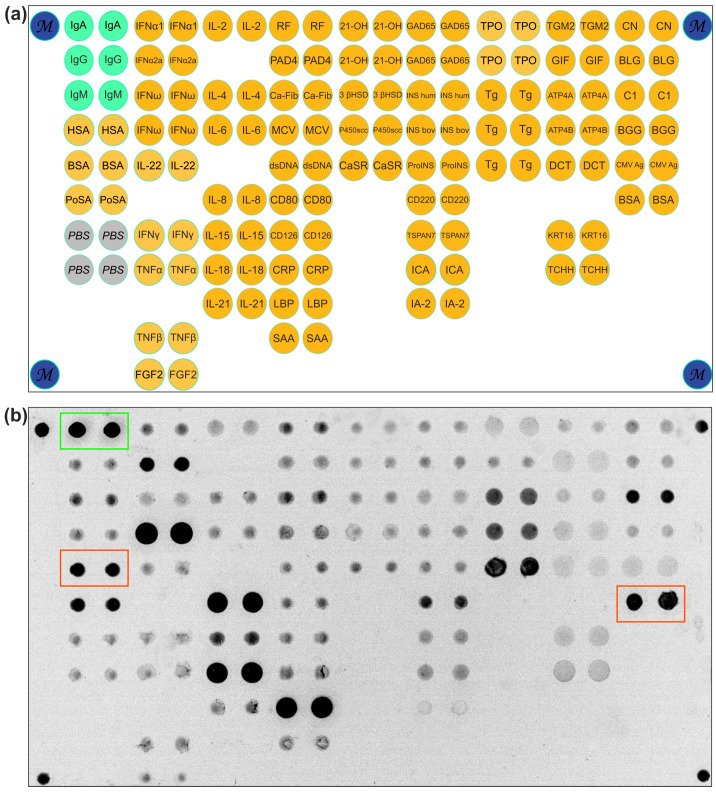
(**a**) The microarray layout. The microarray contained 126 elements with immobilized proteins, six control elements with human immunoglobulins A, G, and M (IgA, IgG, and IgM), four empty hydrogel elements without immobilized proteins (PBS), and four elements with a fluorescent marker (M). The catalog number and the source for each of the immobilized proteins are listed in Table S1. Abbreviations: IgA—immunoglobulin A; IgG—immunoglobulin G; IgM—immunoglobulin M; HSA—human serum albumin; BSA—bovine serum albumin; PoSA—albumin from porcine serum; PBS—empty gel; IFNa1—interferon alpha; INFa2a—interferon alpha 2a; IFNω—interferon omega; IL-22—interleukin 22; IFNγ—interferon gamma; TNFα—tumor necrosis factor alpha; TNFβ—tumor necrosis factor beta; FGF2—fibroblast growth factor 2; IL-2—interleukin 2; IL-4—interleukin 4; IL-6—interleukin 6; IL-8—interleukin 8; IL-15—interleukin 15; IL-18—interleukin 18; IL-21—interleukin 21; RF—Fc fragment from papain-digested human IgG (heavy chain dimer); PAD4—peptidylarginine deiminase 4; Ca-Fib—carbamylated human fibrinogen; MCV—citrullinated vimentin; dsDNA—double-stranded DNA; CD80—cluster of differentiation 80; CD126—sIL-6 receptor α; CRP—C-reactive protein; LBP—lipopolysaccharide-binding protein; SAA—serum amyloid A1; 21-OH—cytochrome P450c21; 3 βHSD—3β-hydroxysteroid dehydrogenase; P450scc—cholesterol side-chain cleavage enzyme; CaSR— Ca-sensing receptor; GAD-65—glutamic acid decarboxylase 65 kDa; INS hum—insulin human; INS bov—insulin bovine; ProINS—proinsulin; CD220—insulin receptor; TSPAN7—tetraspanin-7; ICA—islet cell autoantigen 1; IA-2—tyrosine phosphatase-like autoantigen; TPO—thyroid peroxidase; Tg—thyroglobulin; TGM2—tissue transglutaminase 2; GIF—gastric intrinsic factor; ATP4A—alpha subunit of parietal cell H+/K+-ATPase; ATP4B—beta subunit of parietal cell H+/K+-ATPase; DCT—dopachrome delta-isomerase; KRT16—keratin 16; TCHH—trichohyalin; CN—casein; BLG—b-Lactoglobulin; BGG—bovine γ-globulin; C1—bovine collagen type I. (**b**) A fluorescence image of the microarray obtained on the Cy3 fluorescence channel after the analysis of a serum sample from a celiac disease patient. The green frame highlights positive signals from controls containing immobilized IgA. The red frame highlights positive signals from immobilized BSA.

In this study, serum samples from 150 adult patients were evaluated using a multiplex microarray assay. Indirect immunofluorescence was employed to detect IgA and IgG antibodies against immobilized proteins, with detection carried out via fluorescently labeled anti-human secondary antibodies (anti-IgA-Cy3 and anti-IgG-Cy5.5). The microarray included immobilized antigens such as autoantigens associated with autoimmune diseases, cytokines and inflammatory markers, HSA, BSA, and PoSA (Figure 1a). Antigen–antibody complexes were visualized using a laser microarray analyzer, with Cy3 (green channel) being used for IgA detection and Cy5.5 (red channel) for IgG detection. Figure 1b illustrates a microarray fluorescence image (Cy3 channel) from a celiac disease patient, highlighting IgA antibody reactivity.

**Figure 2:**
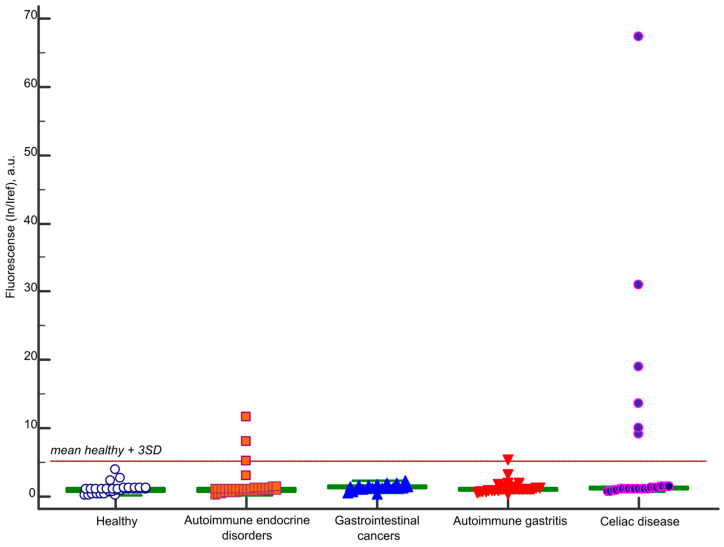
IgA antibodies to bovine serum albumin detected by multiplex microarray analysis in groups of patients with celiac disease, controls, and healthy donors.

Figure 2 presents the immunoassay results for IgA antibodies targeting BSA, detected using microarrays across all study groups. The threshold for a positive result was defined as the mean value derived from samples of 33 healthy individuals plus three standard deviations (I_n_/I_ref_ ≥ 5.2). Ten samples tested positive for IgA antibodies to BSA. Among these, 6/27 (22%) were from the celiac disease group, 1 belonged to a patient in the autoimmune gastritis group (with comorbid autoimmune endocrine disorders), and 3 were from the autoimmune endocrine disorder group. No positive results were detected in the gastrointestinal cancers group or the healthy control group.

A further analysis of the ten highly positive IgA-BSA samples for IgE and IgM antibodies via multiplex microarray assays revealed no detectable antibodies targeting BSA in these classes. BSA (heat shock fraction, ≥98% purity; A7030, Sigma-Aldrich, St. Louis, MO, USA) was used as the primary antigen. To eliminate the potential impact of a particular BSA preparation on the immunoassay results, BSA from an alternate manufacturer (MB083, Himedia, Mumbai, India) was incorporated into the microarray. The results from both BSA preparations demonstrated comparable outcomes.

A trend toward age specificity was observed among patients with IgA antibodies to BSA, with seven of ten positive cases occurring in individuals aged 18–30 years (Figure S1). However, no significant correlation was found between patient age and IgA-BSA antibody levels across all study groups (r = −0.1910, *p* = 0.0209, *n* = 150).

**Figure 3:**
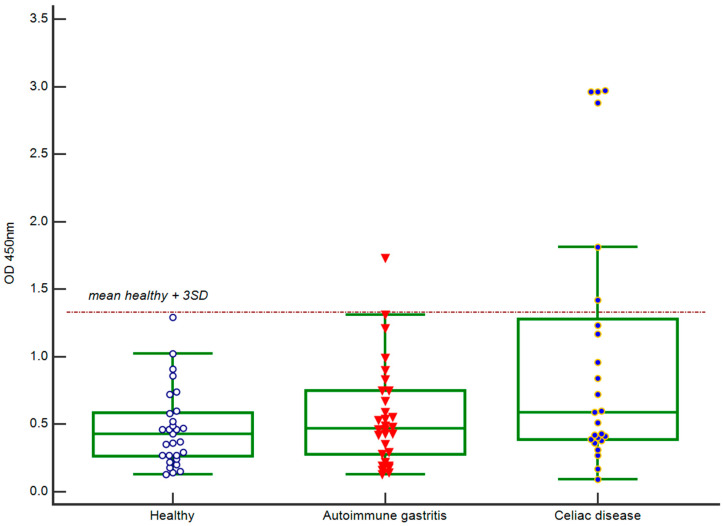
Detection of IgA antibodies to BSA in healthy donors, autoimmune gastritis patients, and celiac disease patients via ELISA.

IgA anti-BSA antibody levels were validated using an in-house ELISA for three cohorts: celiac disease patients (*n* = 27), autoimmune gastritis patients (*n* = 30), and healthy donors (*n* = 33). Additionally, three IgA-BSA-positive samples from the autoimmune endocrine disorders group (identified via microarray) were re-analyzed using an ELISA. The same BSA was used as the immobilized antigen (heat shock fraction, ≥98% purity; A7030, Sigma-Aldrich, St. Louis, MO, USA). Figure 3 presents the ELISA results for IgA antibodies targeting BSA for healthy donors, autoimmune gastritis patients, and celiac disease patients. The threshold for positivity was defined as the mean optical density at 450 nm (OD450) of 33 healthy donors plus three standard deviations (OD450 ≥ 1.32). All samples negative in the multiplex microarray assay were also negative in the ELISA. All samples positive in the multiplex microarray assay showed complete concordance in the ELISA.

For patients with celiac disease, the levels of IgA antibodies to tissue transglutaminase, gliadin, and total IgA levels were determined using commercially available ELISA kits. Patients from other cohorts that were positive for IgA-BSA were also examined. No elevation in anti-TGM2 IgA levels was observed in any participant. Elevated anti-gliadin IgA (63.0 U/mL; normal < 25 U/mL) was detected in a sample from a 59-year-old male with celiac disease and autoimmune thyroiditis. Notably, this patient tested negative for IgA-BSA. A 33-year-old male with celiac disease exhibited IgA deficiency (total IgA < 0.06 g/L), with no detectable IgA-BSA.

Table 1 summarizes the clinical characteristics of patients with elevated IgA-BSA levels. All ten samples demonstrated normal levels of IgA antibodies to tissue transglutaminase 2 (TG2) (<20 U/mL) and anti-gliadin IgA antibodies (<25 U/mL). The total IgA levels were within the normal reference range (0.9–5.0 g/L) for seven samples. Three samples exhibited marginally subnormal total IgA levels (0.63 g/L for #A248; 0.82 g/L for #A314; and 0.84 g/L for #A315).

Reduced IgA anti-BSA antibody activity was observed with successive freeze–thaw cycles, as demonstrated by multiplex and ELISA analyses. Consequently, only the results from samples subjected to a single freeze–thaw cycle were included in the study. The effects of long-term sample storage at −20 °C or −80 °C on assay performance were not assessed.

### 2.2. Analysis of Isolated Serum Antibodies to BSA

**Figure 4:**
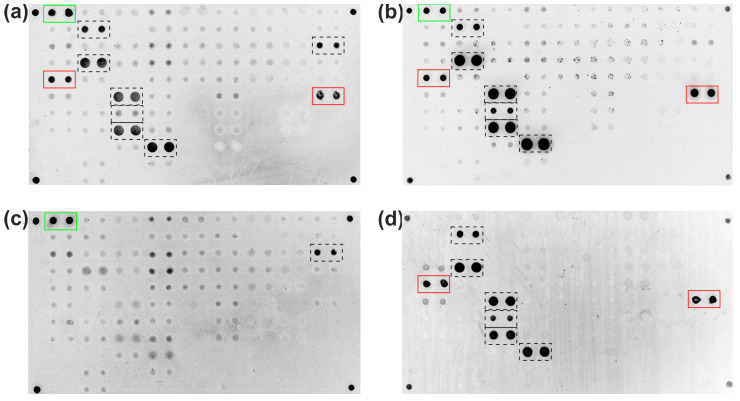
Fluorescent microarray images illustrating the analysis of: (**a**) a serum sample containing IgA-BSA antibodies; (**b**) anti-BSA antibodies isolated from the sample using a BSA-coupled immunosorbent; (**c**) the original sample after BSA pre-absorption to remove anti-BSA antibodies; and (**d**) a direct assay with a fluorescently labeled rabbit polyclonal anti-BSA antibody (control for BSA impurity interference). Green frame: control elements for detecting anti-human IgA-Cy3. Red frame: elements with immobilized BSA. Dotted-line frame: additional positive signals.

IgA antibodies targeting BSA were isolated from serum samples of high-titer patients using a magnetic bead-based immunosorbent with immobilized BSA. These purified antibodies were then subjected to multiplex microarray analysis, which included 66 proteins such as cytokines, acute-phase proteins, autoantigens, and cow’s milk proteins (Table S1). The obtained fluorescence images (Figure 4b) were then compared with the results of the analysis of the original serum sample (Figure 4a). Furthermore, a comparison was made with the results of the analysis of the original sample, in which the antibodies binding to BSA were previously removed by immunoprecipitation (Figure 4c). Finally, a direct analysis of fluorescently labeled monoclonal antibodies to BSA was carried out to demonstrate the effect of BSA impurities on the assay results (Figure 4d).

As shown in Figure 4a, the original sample exhibited signals from immobilized BSA sourced from two distinct manufacturers (highlighted by a red frame) in addition to control elements for detecting anti-human IgA-Cy3 (green frame). Additional signals were observed from immobilized proteins, including interferon alpha 2a, one of two immobilized omega interferons, interleukin 8, interleukin 15, interleukin 18, lipopolysaccharide-binding protein, and bovine collagen type I (highlighted by a dotted-line frame). Following the isolation of the BSA-binding antibody fraction from the serum sample via an immunosorbent, a microarray analysis of the purified fraction revealed the retention of the aforementioned binding profile with the exception of no binding to immobilized bovine collagen type I (Figure 4b). Following the removal of BSA-binding antibodies from serum via immunoprecipitation (Figure 4c), signals only persisted for immobilized bovine collagen type I (aside from control elements). Interference in the immunoassay caused by anti-BSA antibodies was finally confirmed by direct analysis with fluorescently labeled rabbit polyclonal antibodies to BSA (Figure 4d).

No cross-reactivity was observed between serum IgA antibodies targeting BSA and immobilized proteins confirmed to lack BSA impurities. As shown in Figure 4b,d, the isolated BSA-specific antibody fraction showed no binding to proteins unreactive to rabbit polyclonal anti-BSA antibodies. IgA antibodies to BSA isolated from the sera of celiac disease patients did not cross-react with immobilized HSA but demonstrated binding to PoSA in some samples.

### 2.3. IgA and IgG Antibody Repertoire to Serum Albumins

**Figure 5:**
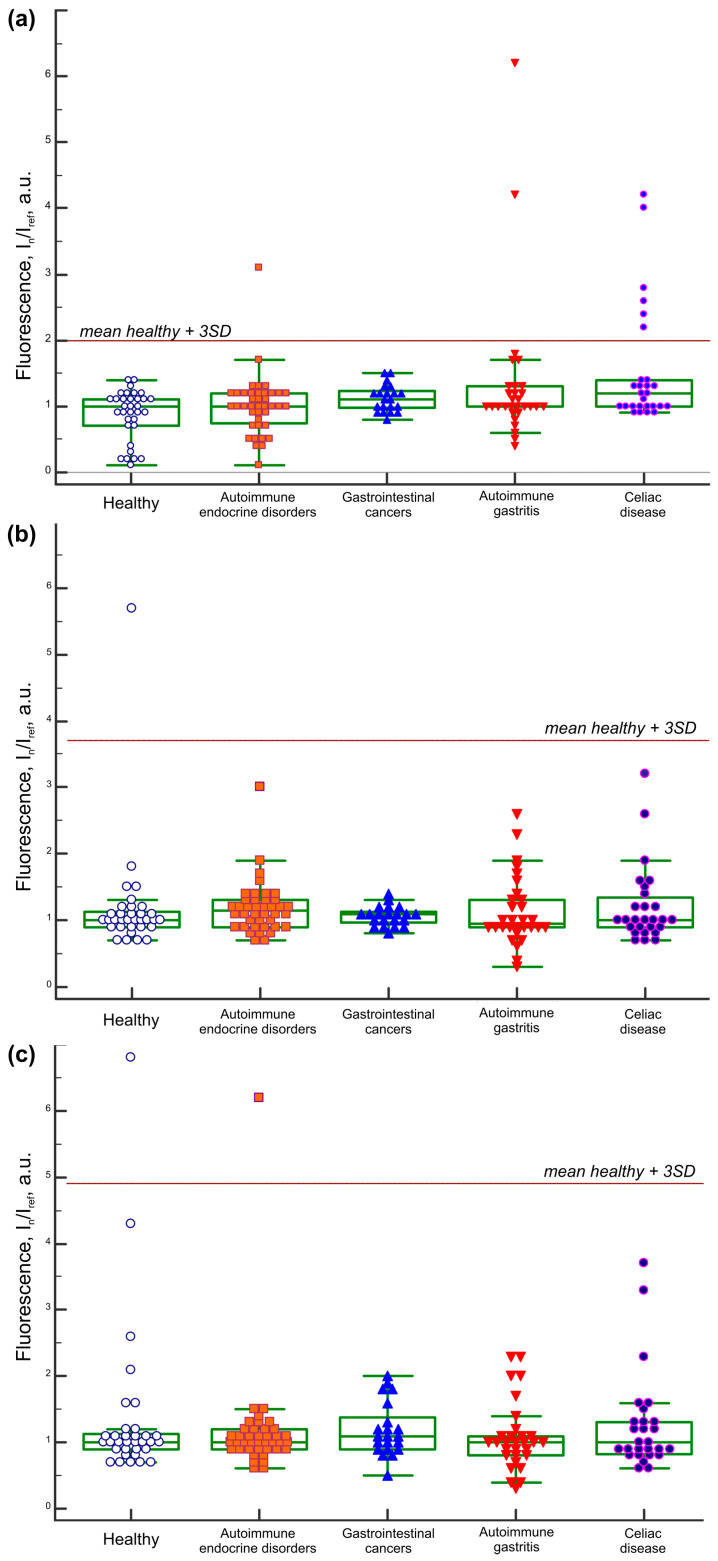
Antibody reactivity profiles detected via microarray multiplex assay: (**a**) IgA antibodies targeting porcine serum albumin (PoSA); (**b**) IgG antibodies targeting PoSA; (**c**) IgG antibodies targeting BSA.

Albumin from human serum (A1653, Sigma-Aldrich, St. Louis, MO, USA) and albumin from porcine serum (A1830, Sigma-Aldrich, St. Louis, MO, USA) were used to detect IgA and IgG antibodies against different serum albumins, which are highly homologous proteins to BSA. The results of the multiplex analysis of sera from 150 adult patients are presented in Figure 5. The cutoff values were determined as the mean plus three standard deviations of signals from samples of 33 healthy subjects, expressed in arbitrary units. Accordingly, the cut-off values for the microarray analysis were as follows: IgA-HSA ≥ 2.0, IgA-BSA ≥ 5.2, IgA-PoSA ≥ 2.0, IgG-HSA ≥ 1.7, IgG-BSA ≥ 4.9, and IgG-PoSA ≥ 3.7.

IgA and IgG antibodies to HSA were undetectable in all analyzed samples. Data for IgA antibodies to BSA are shown in Figure 2. Both IgA and IgG class antibodies to porcine serum albumin detected using a multiplex microarray analysis in groups of celiac disease patients, controls, and healthy donors are presented in Figure 5a and Figure 5b, respectively. IgG antibodies to BSA are shown in Figure 5c.

No significant correlations were observed between the levels of IgA antibodies to BSA and IgG antibodies to BSA (r = 0.3346, *p* < 0.0001, *n* = 150), levels of IgA antibodies to BSA and IgA antibodies to PoSA (r = 0.5224, *p* < 0.0001, *n* = 150), and levels of IgA antibodies to PoSA and IgG antibodies to PoSA levels (r = 0.2977, *p* = 0.0002, *n* = 150). A weak correlation was found between the levels of IgG antibodies to BSA and IgG antibodies to PoSA (r = 0.5803, *p* < 0.0001, *n* = 150).

**Figure 6:**
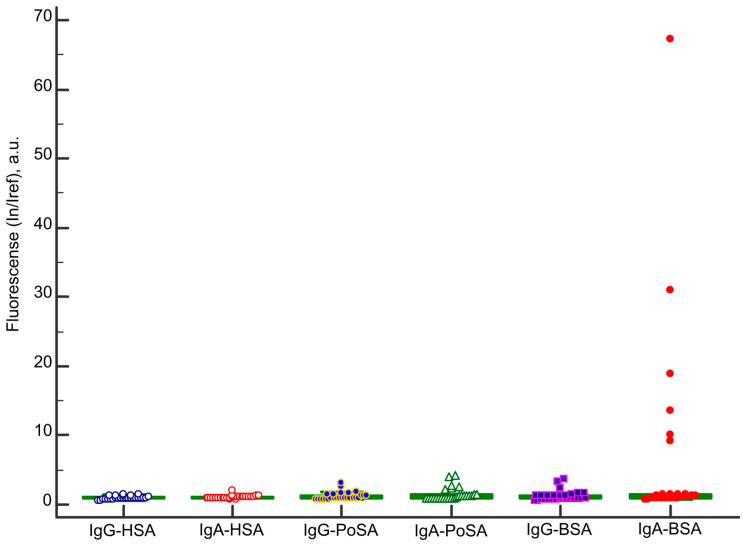
Antibodies to albumins in patients with celiac disease. Abbreviations: IgA—immunoglobulin A; IgG—immunoglobulin G; BSA—bovine serum albumin; PoSA—porcine serum albumin; HSA—human serum albumin.

The repertoire of IgA and IgG antibodies targeting albumins in CD patients is summarized in Figure 6. There were no antibodies to HSA (I_n_/I_ref_ ≤ 2.0). Low positive samples for IgA and IgG antibodies to PoSA and IgG antibodies to BSA (I_n_/I_ref_ ≤ 5.0) were detected. High positive samples for IgA to BSA (I_n_/I_ref_ ≥ 5.2) were revealed in 6 of 27 CD patients.

## 3. Discussion

In a study of 27 celiac disease patients, a multiplex microarray analysis targeting 66 proteins (including bovine, porcine, and human albumins; Figure 1) revealed highly elevated BSA-specific IgA levels in 6 patients (22%). Elevated levels of serum IgA antibodies to BSA in CD patients may reflect persistent mucosal inflammation and damage despite adherence to a GFD, potentially contributing to altered intestinal epithelial permeability. To validate this hypothesis, we analyzed additional cohorts, including patients with autoimmune gastritis, gastrointestinal cancers (gastrointestinal tumors of the stomach and small intestine and patients with colorectal cancer), autoimmune endocrine disorders, and healthy controls (Figure 2). No BSA-specific IgA was detected in healthy individuals or gastrointestinal cancer patients. However, elevated IgA-BSA levels were observed in three patients with autoimmune endocrine disorders and one patient with autoimmune gastritis. These findings were corroborated by an ELISA, with no discrepancies between the two methods (Figure 3).

Notably, only three of the ten patients with elevated BSA-specific IgA levels were over 30 years old (ages 45, 47, and 76). While this age bias warrants consideration, we found no significant correlation between IgA-BSA levels and patient age (r = −0.1910, *p* = 0.0209, *n* = 150) (Figure S1). Previous studies suggest age-dependent variations in BSA-specific antibody responses. Hilger et al. demonstrated that BSA-IgG titers peak in children aged 0–10 years and steadily decline until age 50 [24]. Additionally, the amount of intact BSA entering circulation may differ between children and adults due to developmental differences in gastrointestinal barrier function and gastric pH (pH = 3–4 in children vs. pH = 2 in adults) [18]. Kemp et al. reported elevated BSA-specific IgA1 levels (but not IgA2) in 7 of 11 celiac children on a gluten-free diet compared to healthy controls (30 adults and 26 children) [21]. For 6 of these 11 children (2–13 years old), serum samples were collected after a gluten challenge for three months: four of six samples also had elevated levels of IgA1 antibodies to BSA. Combined with our findings in adult celiac patients, the age-independent nature of IgA-BSA production in CD patients suggests a systemic immune response to dietary antigens, potentially reflecting persistent mucosal inflammation even in GFD-adherent individuals.

In this study, ten patients in the cohort (*n* = 150) exhibited elevated BSA-specific IgA levels above the reference cut-off. Of these, six were diagnosed with celiac disease without concurrent autoimmune conditions (Table 1). The remaining four patients, without a CD diagnosis, presented with combinations of autoimmune diseases and comorbid pathologies. Notably, all four patients had autoimmune thyroiditis alongside type 1 diabetes (T1D) or Addison’s disease:

- A 19-year-old male (#A093) with T1D and autoimmune thyroiditis;
- A 47-year-old female (#A248) with Addison’s disease and autoimmune thyroiditis;
- A 30-year-old male with T1D, autoimmune thyroiditis, autoimmune alopecia, enamel hypoplasia, and Raynaud’s syndrome (#A347);
- A 45-year-old female (#A162) with Addison’s disease, autoimmune thyroiditis, autoimmune gastritis, and vitiligo.

Two of these patients (#A093 and #A162) exhibited fluorescence values in the borderline range (I_n_/I_ref_ = 5.2) but were confirmed positive by an ELISA. A multiplex analysis revealed that serum samples with elevated IgA-BSA levels could contain BSA-specific IgA as the sole anti-albumin antibody or in combination with BSA-specific IgG and/or IgA/IgG antibodies to porcine serum albumin. Importantly, no antibodies to HSA were detected. Additionally, patients with high IgA-BSA levels showed no detectable IgE or IgM antibodies to BSA. All ten patients had normal levels of IgA antibodies against tissue transglutaminase 2 and gliadin with no evidence of total IgA deficiency or elevation.

Antibodies to BSA were isolated from serum samples of patients with elevated BSA-specific IgA levels using an immunosorbent with BSA and analyzed via multiplex assay. By comparing the immunoassay results of the original sample (Figure 4a), the isolated antibody fraction (Figure 4b), the sample obtained post-immunoprecipitation of BSA antibodies (Figure 4c), and a direct analysis of rabbit anti-BSA antibodies (Figure 4d), several conclusions were drawn. First, high levels of BSA-specific IgA can cause interference in antibody assays. Second, antibody fractions isolated using the BSA immunosorbent did not interact with proteins immobilized on a microarray containing no BSA, including thyroid autoantigens (thyroid peroxidase and thyroglobulin), pancreatic β-cell autoantigens (glutamic acid decarboxylase 65 kDa, human insulin, bovine insulin, proinsulin, insulin receptor, tetraspanin-7, islet cell autoantigen 1, and tyrosine phosphatase-like autoantigen), and adrenal-specific autoantigens (steroid 21-hydroxylase) (Figure 4b). Also, isolated BSA-specific IgA did not cross-react with HSA but exhibited binding to PoSA after concentration. Investigating common epitopes shared by bovine and porcine albumin that are targeted by IgA antibodies against BSA requires the use of additional methodologies, such as spectroscopy, chromatography, and molecular docking [25,26,27].

No interaction was observed between BSA-specific IgA and other milk proteins immobilized on the microarray, including casein, bovine β-lactoglobulin, and γ-globulin. Prior studies have shown that bovine casein triggers mucosal inflammation in CD patients adhering to a GFD [28] and is recognized by antigliadin IgA antibodies [29]. At the same time, antigliadin antibody immunoreactivity to BSA was not detected. Our patients did not have antigliadin antibodies, and we also did not observe any cross-reactions of IgA-BSA with casein or gliadin. Furthermore, an ELISA confirmed the absence of cross-reactivity between IgA-BSA and gliadin or transglutaminase 2, as all samples tested negative. Notably, γ-globulin, a milk protein with reported cross-reactive allergenicity to BSA [30], also showed no interaction with BSA-specific IgA in our assays.

Elevated levels of IgG and IgA antibodies to PoSA, as well as IgG antibodies to BSA, were observed across study groups (Figure 5). Notably, healthy individuals may also test positive for these antibodies. Since BSA is commonly used as a stabilizer and carrier protein in vaccines and pharmaceuticals, IgGs response to BSA may reflect individual medical history (e.g., prior vaccinations or therapies) alongside dietary exposure.

No antibodies to HSA were detected in any cohort, including in patients with autoimmune endocrine disorders, despite the high sequence homology (up to 80%) between human and bovine albumins. This contrasts with prior findings where IgG antibodies to HSA were identified in 22 of 180 systemic lupus erythematosus patients and in 13 of 188 healthy controls [31]. In contrast, our healthy cohort showed no detectable HSA antibodies. As demonstrated in Figure 6, no IgA or IgG antibodies to HSA were detected in CD patients. Furthermore, IgA/IgG antibodies to PoSA and IgG antibodies to BSA were negligible compared to the markedly elevated levels of BSA-specific IgA. The clinical significance of detecting these antibodies at high levels in some celiac disease patients remains unclear.

BSA is widely utilized in laboratory settings as a stabilizer, carrier protein, and blocking agent in immunoassays such as ELISA, Western blot, ELISpot, and multiplex arrays. In protein microarrays, hundreds of distinct proteins are often immobilized on a single slide for high-throughput analysis. As demonstrated in Figure 4, the presence of anti-BSA antibodies in serum samples can generate nonspecific binding signals, leading to the misinterpretation of antibody reactivity data. This underscores the importance of accounting for BSAs ubiquity in assay design and validation.

The interference of anti-BSA antibodies and BSA contaminants in immunoassays is well documented. For instance, anti-BSA antibodies and residual BSA in assay components have been shown to skew results in radiobinding assays for insulin autoantibodies [32]. Similarly, removing BSA-reactive antibodies from serum improved the accuracy of IgM quantification for diagnosing acute hepatitis E virus infection [33]. In rheumatoid arthritis patients, anti-BSA antibodies have been shown to interfere with ELISA results when BSA is used as a blocking agent [34]. Elevated levels of polyreactive IgG were found in untreated autoimmune hepatitis patients, including polyreactivity against blocking reagents and the highest binding for BSA [35]. Notably, false positive results due to BSA exposure extend beyond assays: a case study documented a false positive anti-glomerular basement membrane antibody test caused by a BSA-containing surgical adhesive [36]. To mitigate these issues, strategies such as alternative blocking reagents and improved antigen purification have been proposed [37].

Low-titer antibodies to BSA are detectable across diverse patient cohorts and healthy individuals (Figure 5), with some cases exhibiting markedly elevated levels (Figure 2; Table 1). To minimize cross-reactivity and interference in indirect immunoassays utilizing anti-species detection antibodies, blocking and assay buffers devoid of extraneous proteins (e.g., BSA and casein) should be used. The assay should also include sample-specific controls such as antigen-uncoated wells (for ELISA [38,39]) or blank elements (for multiplex assays [40]) to account for nonspecific binding. For multiplex platforms, normalizing signals against blank elements and including controls with known interfering proteins (e.g., BSA) are critical to differentiate specific antibody binding from nonspecific background reactivity. These strategies improve assay accuracy, reduce variability between platforms, and mitigate false-positive/negative results caused by polyreactive antibodies or residual assay contaminants.

This study has several limitations. First, the celiac disease cohort comprised only 27 patients, limiting statistical power. Additionally, non-compliant GFD patients were excluded, potentially biasing the results. Some patients in the celiac disease and autoimmune gastritis groups had comorbid autoimmune endocrine disorders, complicating data interpretation. Patients with inflammatory bowel disease were not included, limiting insights into shared immunological mechanisms. Heterogeneity in IgA-BSA antibody measurements may arise from differences in the prior consumption of BSA-containing products and sample storage conditions (e.g., freeze–thaw cycles and long-term storage at −20 °C/−80 °C).

## 4. Materials and Methods

### 4.1. Study Population

This study was conducted in accordance with the Declaration of Helsinki and approved by the local ethics committee of the Endocrinology Research Centre, Ministry of Health of Russia, Moscow, Russia (protocol No. 14 and date of approval 29 July 2022). A total of 150 adult patients were enrolled. The exclusion criteria included pregnancy or lactation; acute infections or the exacerbation of chronic diseases; severe, life-threatening conditions, such as the decompensation of chronic heart failure, chronic kidney disease (stage C3b or higher), and pulmonary or hepatic insufficiency; immune system pathologies, including congenital or acquired immunodeficiencies and hypersensitivity reactions during the study; recent immunomodulatory therapy (e.g., interleukins, interferons, immunoglobulins, immunosuppressants, cytostatics) within one month prior to enrollment; and vaccinations or revaccinations within one month prior to enrollment (verified via medical documentation). Serum samples were collected from patients, aliquoted, and stored at −20 °C (short-term) or −80 °C (long-term).

The study cohort comprised the following groups: celiac disease patients, autoimmune gastritis patients, gastrointestinal tumor patients (including patients with gastrointestinal stromal tumors and colorectal cancer), autoimmune endocrine disorder patients (e.g., type 1 diabetes, autoimmune thyroiditis, Graves’ disease, Addison’s disease, or combinations thereof), and healthy controls. Detailed patient characteristics are provided in Table 2.

The diagnosis of celiac disease was confirmed through a comprehensive evaluation, including clinical presentation consistent with CD, elevated serological markers (levels of IgA and IgG antibodies to tissue transglutaminase above reference values), and histopathological findings, including hyperregenerative atrophy of the small intestine (Marsh–Oberhuber classification). The diagnostic criteria adhered to the All-Russian Consensus on the Diagnosis and Treatment of Celiac Disease in Adults and Children [41]. At enrollment, all CD patients were adhering to a gluten-free diet.

### 4.2. Microarray Manufacturing

Antibody profiling was performed using hydrogel-based low-density microarrays [42]. Each microarray slide contained 66 immobilized proteins (including 59 unique proteins and 7 variants from different manufacturers), spotted in duplicate. The protein panel included autoantigens associated with autoimmune diseases, cytokines and inflammatory markers, and control elements (see Table S1 for full details). Among the proteins immobilized on the microarray were the following albumins: BSA, heat shock fraction (A7030, Sigma-Aldrich, St. Louis, MO, USA); BSA (MB083, Himedia, Mumbai, India); PoSA, albumin from porcine serum (A1830, Sigma-Aldrich, St. Louis, MO, USA); and HSA, albumin from human serum (A1653, Sigma-Aldrich, St. Louis, MO, USA). The microarray layout is illustrated in Figure 1a.

### 4.3. Detection of Antibodies by Microarrays

IgA and IgG antibodies targeting 66 immobilized proteins were detected using a previously developed multiplex immunoassay [40]. Microarrays were blocked with 1% polyvinyl alcohol (PVA, Sigma-Aldrich, St. Louis, MO, USA) in phosphate-buffered saline (PBS, pH 7.4, Sigma-Aldrich, St. Louis, MO, USA) at room temperature (RT) for 1 h. The samples and antibodies were diluted in 0.14% PVA and 0.14% polyvinyl pyrrolidone (PVP, Sigma-Aldrich, St. Louis, MO, USA) in PBS. An amount of 100 µL of diluted sample (1:62.5) was applied on the microarrays. After incubation with the sample (for two hours at 37 °C) and intermediate washing (PBS with 0.1% Tween 20 for 20 min), binding was revealed with a mixture of anti-human IgG-Cy5.5 and anti-human IgA-Cy3 (2.5 μg/mL each; 50 μL) (Table S2). After overnight incubation at 37 °C, the microarrays were washed with PBS containing 0.1% Tween 20 for 30 min, rinsed, and dried.

Fluorescence images of microarrays were obtained using a proprietary laser-excited microarray analyzer [43]. Signal quantification was performed with proprietary software. For each group of *n* elements containing identical antigens, the resulting signal (I_n_) was calculated as the mean fluorescence intensity of the two corresponding spots. To normalize for variations in the total IgG concentration, the individual background signal from empty gel elements (I_n_/I_ref_) was incorporated into the analysis for each sample. Each high-positive sample was reanalyzed on four separate microarrays, each developed with fluorescently labeled anti-human secondary antibodies targeting IgG, IgA, IgM, and IgE. Details of the secondary antibodies are provided in Table S2.

### 4.4. Direct Assay for BSA Detection and Cross-Reactivity Check

Cy5.5-conjugated rabbit polyclonal anti-BSA antibodies (Thermo Fisher Scientific, Waltham, MA, USA) were used for the direct detection of albumins. An amount of 100 µL of diluted antibodies (2.5 μg/mL) was applied to each microarray. After two hours of incubation at 37 °C, the microarrays were washed with PBS containing 0.1% Tween 20 for 30 min, rinsed, and dried. Fluorescence images were acquired using the same protocol described previously.

### 4.5. Detection of Antibodies by ELISA

#### 4.5.1. TGM2, Anti-Gliadin, and Total IgA

Commercially available ELISA kits (Xema Co., Ltd., Moscow, Russia) were used to quantify anti-tissue transglutaminase 2 IgA antibodies, anti-gliadin IgA antibodies, and total IgA in the serum samples. All assays were performed according to the manufacturer’s instructions.

#### 4.5.2. IgA-BSA

The levels of IgA antibodies to BSA were quantified using an in-house ELISA protocol. Approximately 96-well microplates were coated with 100 µL of BSA (10 µg/mL) (A7030, Sigma-Aldrich, St. Louis, MO, USA) in 0.05 M carbonate–bicarbonate buffer (pH 9.6). The microplates were incubated at 4 °C overnight. The wells were blocked with EveryBlot Blocking Buffer (Bio-Rad Laboratories, Hercules, CA, USA) at 37 °C for 30 min. An amount of 100 µL of serum samples (1:40 dilution) in PBS containing 0.05% Tween-20 was added to the wells and incubated at 37 °C for 1 h. Next, 100 µL of goat anti-human IgA (0.5 µg/mL) (A24460, Invitrogen, Thermo Fisher Scientific, Waltham, MA, USA) was added to the wells and incubated at 37 °C for 30 min. After that, 100 µL of rabbit anti-goat IgG-HRP (0.2 µg/mL) (S0010, Affinity Biosciences, Cincinnati, OH, USA) was added and incubated at 37 °C for 30 min. Subsequently, 100 µL of tetramethylbenzidine substrate (Abisense, Sirius, Russia) was added to each well and incubated in the dark at room temperature for 15 min. The reaction was stopped with 50 µL of 1M HCl. Optical density was measured immediately at 450 nm using a HiPo MPP-96 microplate photometer (Biosan, Riga, Latvia). Between each incubation step, the wells were washed at least three times with PBS containing 0.05% Tween-20.

### 4.6. Isolation of BSA-Specific Antibodies

BSA was covalently immobilized on amino-functionalized magnetic nanoparticles (iron oxide, 300–400 nm; Sileks, Moscow, Russia) using glutaraldehyde crosslinking following the manufacturer’s protocol. In brief, 100 μL of magnetic particle solution (5 mg/mL) was washed, resuspended in 250 μL of PBS, and 250 μL of 25% glutaraldehyde was added and incubated for 3 h with gentle rocking. After four washes with PBS, the particles were resuspended in 500 µL of PBS containing 0.01% Tween20 (PBSt) and supplemented with 100 µL of BSA (0.2 mg/mL) (A7030, Sigma-Aldrich, St. Louis, MO, USA), incubated overnight with gentle rocking, washed four times with PBS again, and resuspended in 100 µL of PBSt.

The antibodies were isolated from 20 µL of blood serum using 50 µL of magnetic particle suspension with immobilized BSA. Magnetic particles were blocked for 1 h in 100 μL of EveryBlot Blocking Buffer (Bio-Rad Laboratories, Hercules, CA, USA), washed, and resuspended in 500 μL of PBSt; then, a blood serum sample (20 μL) was added, incubated for 1 h with gentle rocking, washed three times with 500 μL of PBSt, and resuspended in 100 μL of PBSt. An amount of 100 μL of Elution Buffer (0.1 M Gly-HCL, pH 2.6) was added to the particles, and they were incubated for 10 min with periodical mixing by pipetting. The eluate was collected and immediately neutralized with 20 μL of 1 M Tris-HCl (pH 8.5).

### 4.7. BSA Antibody Immunoprecipitation

Serum anti-BSA antibody removal was performed as described previously [34]. An amount of 20 μL of blood serum sample was diluted 10-fold with PBS, and 56 μL of BSA (50 mg/mL) was added. The mixture was incubated for 1 h at 37 °C, followed by overnight incubation at 4 °C. The samples were centrifuged at 10,000× *g* for 20 min at 4 °C.

### 4.8. Data Analysis

Statistical analysis was performed using MedCalc Statistical Software version 20.008 (MedCalc Software bv, Ostend, Belgium). Pearson’s correlation coefficient was used to assess correlations between datasets, with *p* < 0.05 being considered statistically significant.

The cutoff values for antibodies to albumins were determined as three standard deviations above the mean of healthy individuals. For the ELISA, the cut-off level for IgA to BSA was calculated as OD450nm ≥ 1.32. For the microarray analysis, the cut-off values were calculated as follows: IgA: HSA ≥ 2.0, BSA ≥ 5.2, and PoSA ≥ 2.0; IgG: HSA ≥ 1.7, BSA ≥ 4.9, and PoSA ≥ 3.7.

## 5. Conclusions

Antibodies targeting bovine serum albumin are prevalent across both healthy individuals and various patient populations. Elevated titers of IgG and IgA anti-BSA antibodies may interfere with immunoassays where BSA is utilized as a stabilizer, carrier protein, or blocking reagent. A subset of adult celiac disease patients on a gluten-free diet, despite being seronegative for IgA antibodies to tissue transglutaminase 2 and gliadin, exhibit high IgA reactivity to BSA. These antibodies may serve as biomarkers of persistent intestinal mucosal inflammation in a subgroup of GFD-adherent patients or contribute to ongoing clinical symptoms. There is a need for further investigation of the temporal dynamics of anti-BSA antibodies in celiac disease patients before and during GFD implementation. Expanding patient cohorts to include inflammatory bowel disease subtypes, such as Crohn’s disease and ulcerative colitis, holds strategic value for investigating shared immunological mechanisms.

## Figures and Tables

**Table 1:** Characteristics of patients with elevated levels of IgA-BSA. Abbreviations: f—female; m—male; IgA—immunoglobulin A; IgG—immunoglobulin G; BSA—bovine serum albumin; PoSA—porcine serum albumin; cut-off—three standard deviations from average value of healthy individuals.

Sample ID	Age	Gender	Diagnosis	Microarray		ELISA
				IgA-BSA, I_n_/I_ref_, Cut-Off ≥ 5.2	Other Anti-Albumins Antibodies	IgA-BSA, OD 450 nm, Cut-Off ≥ 1.32
A093	19	m	Type 1 diabetes mellitus, autoimmune thyroiditis	5.2	-	1.46
A248	47	f	Addison’s disease, autoimmune thyroiditis	8.0	IgA-PoSA	2.11
A347	30	m	Type 1 diabetes mellitus, autoimmune thyroiditis, autoimmune alopecia, dental enamel hypoplasia, Raynaud’s syndrome	11.7	IgG-BSA	1.99
A162	45	f	Autoimmune gastritis, Addison’s disease, autoimmune thyroiditis, vitiligo	5.2	IgA-PoSA	1.73
A113	18	m	Celiac disease	30.9	IgA-PoSA	1.81
A268	76	f	Celiac disease	13.6	IgA-PoSA	2.88
A308	29	m	Celiac disease	9.1	-	1.42
A314	18	m	Celiac disease	18.9	IgA-PoSA	2.97
A315	30	m	Celiac disease	10.0	IgA-PoSA	2.96
A321	26	f	Celiac disease	67.3	IgG-BSA IgA-PoSA	2.96

**Table 2:** Patient characteristics.

Group	*n*	Mean Age (Range)	Female, %	Male, %
Celiac disease	27	39 (18–76)	63	37
Autoimmune gastritis	30	51.5 (27–86)	93	7
Gastrointestinal cancers	21	61 (31–80)	44	56
Autoimmune endocrine disorders	39	44 (19–81)	77	23
Healthy	33	39 (19–67)	76	24
Total	150	-	-	-

## Data Availability

The authors confirm that the data supporting the findings of this study are available within the article and/or its Supplementary Materials.

## Supplementary Materials

The supporting information can be downloaded at: https://www.mdpi.com/article/10.3390/ijms26114988/s1.

## Abbreviations

The following abbreviations are used in this manuscript:

CD	Celiac disease
BSA	Bovine serum albumin
GFD	Gluten-free diet
TGM2	Tissue transglutaminase 2
Ig	Immunoglobulin
T1D	Type 1 diabetes mellitus
HSA	Human serum albumin
PoSA	Porcine serum albumin
Cy	Cyanine dyes
ELISA	Enzyme-linked immunosorbent assay
OD	Optical density
