# Supplementary material for: IgA Antibodies to Bovine Serum Albumin in Adult Patients with Celiac Disease
Source: Int J Mol Sci. 2025 May 22;26(11):4988. doi: 10.3390/ijms26114988 (PMC12154404; doi:10.3390/ijms26114988)
Supplement: Supplementary file 1 [file ijms-26-04988-s001.zip › Table S1. Proteins immobilized on the microarray.pdf]

Table S1. Proteins immobilized on the microarray

Protein	Abbreviation	Catalog #	Source
Marker element	M	-	-
Immunoglobulin A	IgA	31148	Thermo
Immunoglobulin G1	IgG	31R-1087	Biosynth
Immunoglobulin M	igM	16-16-090713	Athens Research
Human serum albumin	HSA	A1653	Sigma
Bovine serum albumin	BSA	A7030	Sigma
Albumin from porcine serum	PoSA	A1830	Sigma
Empty gel	PBS	-	-
Empty gel	PBS	-	-
Interferon alpha 1	IFNa1	228-10814	Raybiotech
Interferon alpha 2a	INFa2a	11100-1	BPL Assay Science
Interferon omega	IFNω	300-02J	PeproTech
Interferon omega	IFNω	BMS304	Thermo
Interleukin 22	IL-22	200-22	PeproTech
Interferon gamma	INFγ	230-00210-10	Raybiotech
Tumor necrosis factor alpha	TNFα	PSG250	Sci-Store
Tumor necrosis factor beta	TNF-β	300-01B	PeproTech
Fibroblast growth factor 2	FGF2	PSG060	Sci-Store
Interleukin 2	IL-2	PSG210	Sci-Store
Interleukin 3	IL-3	PSG160	Sci-Store
Interleukin 4	IL-4	PSG040	Sci-Store
Interleukin 6	IL-6	PSG180	Sci-Store
Interleukin 7	IL-7	PSG240	Sci-Store
Interleukin 8	IL-8	208-IL	RD Systems
Interleukin 15	IL-15	PSG220	Sci-Store
Interleukin 18	IL-18	B001-5	MBL
Interleukin 21	IL-21	PSG260	Sci-Store
Fc fragment from papain-digested human IgG (heavy chain dimer)	RF	ATF01-01	Arotec Diagnostic
Peptidylarginine Deiminase 4	PAD4	10500	Cayman Chemical
Carbamylated Human Fibrinogen	Ca-Fib	21370	Cayman Chemical
Citrullinated Vimentin	MCV	21942	Cayman Chemical
Double stranded DNA	dsDNA	ATD01-10	Arotec Diagnostic
The Cluster of differentiation 80	CD80	6RAY829	LSBio
sIL-6 Receptor α	CD126	ab167742	Abcam
C-reactive protein	CRP	8CR8	HyTest
Lipopolysaccharide binding protein	LBP	870-LP-025/CF	R&D Systems
Serum amyloid A1	SAA	8SA1	HyTest
Cytochrome P450c21	21-OH	ab225641	Abcam
Cytochrome P450c21	21-OH	CSB-EP006400HU	Cusabio

3β-hydroxysteroid dehydrogenase	3 βHSD	MBS718727	MyBioSource
Cholesterol side-chain cleavage enzyme	P450scc	MBS948952	MyBioSource
Ca-sensing receptor	CaSR	CSB-EP004558HU	Cusabio
Glutamic acid decarboxylase 65 kDa	GAD-65	228-20881	Raybiotech
Glutamic acid decarboxylase 65 kDa	GAD-65	H00002572-P03	Abnova
Insulin human	INS hum	ab123768	Abcam
Insulin bovine	INS bov	I-6634	Sigma
Proinsulin	ProINS	1336-PN-050	R&D Systems
Insulin receptor	CD220	1544-IR-050/CF	R&D Systems
Tetraspanin-7	TSPAN7	CSB-EP025165HU	Cusabio
Islet cell autoantigen 1	ICA	CSB-EP010947HU(F1)	Cusabio
Tyrosine phosphatase like autoantigen	IA-2	ab42590	Abcam
Thyroid peroxidase	TPO	8RTPO	HyTest
Thyroid peroxidase	TPO	R131	Xema Co., Ltd
Thyroglobulin	Tg	R132	Xema Co., Ltd
Thyroglobulin	Tg	8TG52	HyTest
Thyroglobulin	Tg	8RTG4	HyTest
Tissue transglutaminase 2	TGM2	4376-TG	R&D Systems
Gastric Intrinsic Factor	GIF	abx166676	Abbexa
Alpha subunit of the parietal cell H+/K+-ATPase	ATP4A	abx065478	Abbexa
Beta subunit of the parietal cell H+/K+-ATPase	ATP4B	CSB-EP002343HUe1	Cusabio
Dopachrome delta-isomerase	DCT	abx166564	Abbexa
Keratin 16	KRT16	abx067610	Abbexa
Trichohyalin	TCHH	abx166773	Abbexa
Casein	CN	C805180	Macklin
b-Lactoglobulin	BLG	L-4756	Sigma
Bovine γ-Globulin	BGG	#5000209	BioRad
Bovine collagen type I	C1	B C11-NCL	Imtek
Cytomegalovirus Pp150 Protein	pp150	RE003	Xema Co., Ltd
BSA	BSA	MB083	Himedia
