# Supplementary material for: IgA Antibodies to Bovine Serum Albumin in Adult Patients with Celiac Disease
Source: Int J Mol Sci. 2025 May 22;26(11):4988. doi: 10.3390/ijms26114988 (PMC12154404; doi:10.3390/ijms26114988)
Supplement: Supplementary file 1 [file ijms-26-04988-s001.zip › Table S2. Fluorescent anti-human conjugates.pdf]

Table S2. Fluorescent anti-human conjugates

Abbreviation	Description	#; Source	Conjugate
anti-h IgA	Goat-anti-human IgA	A24460; Invitrogen	Cy3
anti-h IgG	Goat-anti-human IgG	31163; Invitrogen	Cy5.5
anti-h IgM	Goat-anti-human IgM	A24484; Invitrogen	Cy3
anti-h IgE	Goat anti-Human IgE	A80-108A, Bethyl	Cy5

Cyanine dyes Cy3 (GE HealthCare, USA), Cy5 and Cy5.5 (Lumiprobe, Russia) were used as fluorescent labels. The conjugation reaction of the fluorophore in the form of N-hydroxysuccinimide activated ester with the protein was performed according to the manufacturer's method. Excess fluorophore was removed by gel filtration on chromatographic columns packed with Sephadex G-25 coarse and equilibrated with PBS.
