# Supplementary figures and images for: IgA Antibodies to Bovine Serum Albumin in Adult Patients with Celiac Disease
Source: Int J Mol Sci. 2025 May 22;26(11):4988. doi: 10.3390/ijms26114988 (PMC12154404; doi:10.3390/ijms26114988)

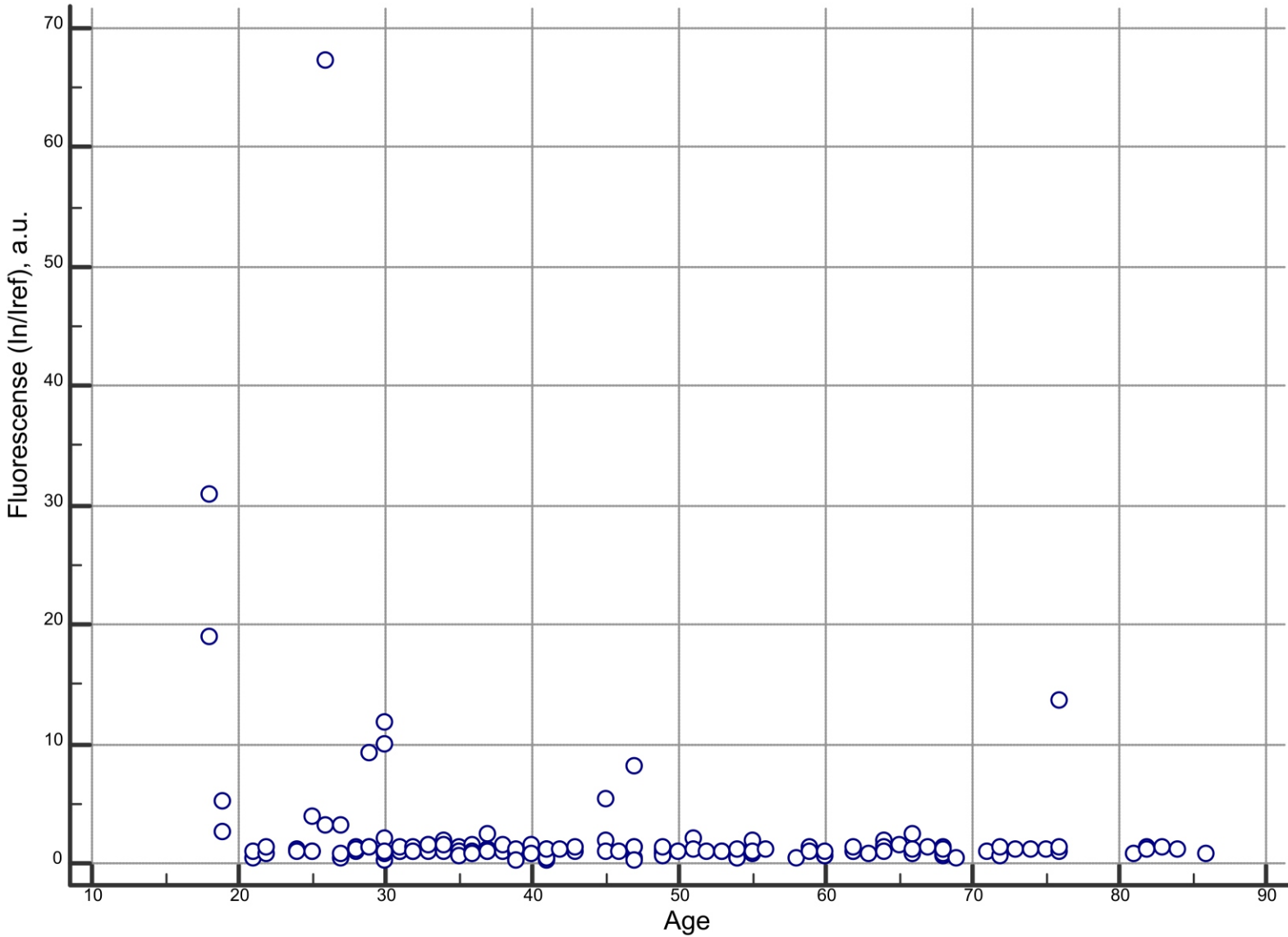

Supplement: Supplementary file 1 [file ijms-26-04988-s001.zip › Figure S1. Level of IgA antibody to BSA in relation to age.pdf]
